# Author Correction: Self-administered mindfulness interventions reduce stress in a large, randomized controlled multi-site study

**DOI:** 10.1038/s41562-024-01947-z

**Published:** 2024-07-09

**Authors:** Alessandro Sparacio, Hans IJzerman, Ivan Ropovik, Filippo Giorgini, Christoph Spiessens, Bert N. Uchino, Joshua Landvatter, Tracey Tacana, Sandra J. Diller, Jaye L. Derrick, Joahana Segundo, Jace D. Pierce, Robert M. Ross, Zoë Francis, Amanda LaBoucane, Christine Ma-Kellams, Maire B. Ford, Kathleen Schmidt, Celia C. Wong, Wendy C. Higgins, Bryant M. Stone, Samantha K. Stanley, Gianni Ribeiro, Paul T. Fuglestad, Valerie Jaklin, Andrea Kübler, Philipp Ziebell, Crystal L. Jewell, Yulia Kovas, Mahnoosh Allahghadri, Charlotte Fransham, Michael F. Baranski, Hannah Burgess, Annika B. E. Benz, Maysa DeSousa, Catherine E. Nylin, Janae C. Brooks, Caitlyn M. Goldsmith, Jessica M. Benson, Siobhán M. Griffin, Stephen Dunne, William E. Davis, Tam J. Watermeyer, William B. Meese, Jennifer L. Howell, Laurel Standiford Reyes, Megan G. Strickland, Sally S. Dickerson, Samantha Pescatore, Shayna Skakoon-Sparling, Zachary I. Wunder, Martin V. Day, Shawna Brenton, Audrey H. Linden, Christopher E. Hawk, Léan V. O’Brien, Tenzin Urgyen, Jennifer S. McDonald, Kim Lien van der Schans, Heidi Blocker, Caroline Ng Tseung-Wong, Gabriela M. Jiga-Boy

**Affiliations:** 1 LIP/PC2s, Université Grenoble Alpes, Grenoble, France; 2 School of Psychology, Swansea University, Swansea, UK; 3 Singapore Institute for Clinical Sciences (SICS), A*STAR, Singapore, Singapore; 4 Annecy Behavioral Science Lab, Saint-Jorioz, France; 5 Institut Universitaire de France, Paris, France; 6 Institute of Psychology, Czech Academy of Sciences, Prague, Czech Republic; 7 Faculty of Education, Institute for Research and Development of Education, Charles University, Prague, Czech Republic; 8 Centre of Social and Psychological Sciences, Slovak Academy of Sciences, Štefánikova, Bratislava, Slovakia; 9 Department of Economics, Management and Statistics (DEMS), University of Milano-Bicocca, Milan, Italy; 10 Spiessens Coaching Solutions Ltd, Manchester, UK; 11 Department of Psychology, College of Social and Behavioral Science, University of Utah, Salt Lake City, UT USA; 12 Private University Seeburg Castle, Seekirchen am Wallersee, Austria; 13 LMU Munich, Munich, Germany; 14 University of Houston, Houston, TX USA; 15 School of Psychology, Macquarie University, Sydney, New South Wales Australia; 16 University of the Fraser Valley, Abbotsford, British Colombia Canada; 17 San Jose State University, San José, CA USA; 18 Psychology department, Loyola Marymount University, Los Angeles, CA USA; 19 Ashland University, Ashland, OH USA; 20 SUNY Brockport, Brockport, NY USA; 21 Department of Mental Health, Bloomberg School of Public Health, Johns Hopkins University, Baltimore, MD USA; 22 Australian National University, Canberra, Australian Capital Territory Australia; 23 School of Law and Justice, The University of Southern Queensland, Ipswich, Queensland Australia; 24 University of North Florida, Jacksonville, FL USA; 25 Department of Psychology, University of Würzburg, Würzburg, Germany; 26 Iowa State University, Ames, IA USA; 27 Goldsmiths University of London, London, UK; 28 Pennsylvania Western University California, California, PA USA; 29 University of Konstanz, Konstanz, Germany; 30 Springfield College, Springfield, MA USA; 31 Department of Social and Behavioral Sciences, Glendale Community College, Glendale, AZ USA; 32 St. Olaf College, Northfield, MN USA; 33 University of Limerick, Limerick, Ireland; 34 Northumbria University, Newcastle upon Tyne, UK; 35 Wittenberg University, Springfield, OH USA; 36 University of Edinburgh, Edinburgh, UK; 37 University of California, Merced, Merced, CA USA; 38 University of Southern Indiana, Evansville, IN USA; 39 Pace University, New York, NY USA; 40 Toronto Metropolitan University (formerly Ryerson), Toronto, Ontario Canada; 41 Wayne State University, Detroit, MI USA; 42 Memorial University of Newfoundland, St. John’s, Newfoundland Canada; 43 Centre for Research in Autism and Education, Institute of Education, University College London, London, UK; 44 Department of Psychology and Counselling, The Open University, Milton Keynes, UK; 45 DigiPen Institute of Technology, Redmond, WA USA; 46 University of Canberra, Canberra, Australian Capital Territory Australia; 47 University of Northern Iowa, Cedar Falls, IA USA; 48 Idaho State University, Pocatello, ID USA; 49 Behavioural Science Institute, Radboud University, Nijmegen, the Netherlands; 50 Eastern Oregon University, La Grande, OR USA

**Keywords:** Human behaviour, Psychology

Correction to: *Nature Human Behaviour* 10.1038/s41562-024-01907-7, published online 11 June 2024.

In the version of the article initially published, affiliation 28 included the wrong city and has now been amended to read “Pennsylvania Western University California, California, PA, USA”. Affiliation 31 included the wrong state and has now been corrected to read “Department of Social and Behavioral Sciences, Glendale Community College, Glendale, AZ, USA”. In the “Simulation of the sequential Bayesian design” section of the Methods, “20” has been corrected to “720” in the text “…(translating to a minimum 420 and maximum 720 participants per condition)” and “0.526” has been corrected to “526” in the text “…with the average *n* at the stopping point (BF boundary and maximum *n*) at 526”. These corrections have been made to the HTML and PDF versions of the article.

