# Supplementary material for: Self-administered mindfulness interventions reduce stress in a large, randomized controlled multi-site study
Source: Nat Hum Behav. 2024 Jun 11;8(9):1716–25. doi: 10.1038/s41562-024-01907-7 (PMC11420060; doi:10.1038/s41562-024-01907-7)
Supplement: Supplementary file 1 — Reporting Summary [file 41562_2024_1907_MOESM1_ESM.pdf]

nature portfolio

Corresponding author(s): Alessandro Sparacio, Hans IJzerman, Gabriela Jiga-Boy, Ivan Ropovik

Last updated by author(s): Apr 24, 2024

Reporting Summary

Nature Portfolio wishes to improve the reproducibility of the work that we publish. This form provides structure for consistency and transparency in reporting. For further information on Nature Portfolio policies, see our Editorial Policies and the Editorial Policy Checklist.

Statistics

For all statistical analyses, confirm that the following items are present in the figure legend, table legend, main text, or Methods section.

n/a	Confirmed	
☐	☒	The exact sample size (*n*) for each experimental group/condition, given as a discrete number and unit of measurement
☐	☒	A statement on whether measurements were taken from distinct samples or whether the same sample was measured repeatedly
☐	☒	The statistical test(s) used AND whether they are one- or two-sided<br>*Only common tests should be described solely by name; describe more complex techniques in the Methods section.*
☐	☒	A description of all covariates tested
☐	☒	A description of any assumptions or corrections, such as tests of normality and adjustment for multiple comparisons
☐	☒	A full description of the statistical parameters including central tendency (e.g. means) or other basic estimates (e.g. regression coefficient) AND variation (e.g. standard deviation) or associated estimates of uncertainty (e.g. confidence intervals)
☐	☒	For null hypothesis testing, the test statistic (e.g. *F*, *t*, *r*) with confidence intervals, effect sizes, degrees of freedom and *P* value noted<br>*Give P values as exact values whenever suitable.*
☐	☒	For Bayesian analysis, information on the choice of priors and Markov chain Monte Carlo settings
☐	☒	For hierarchical and complex designs, identification of the appropriate level for tests and full reporting of outcomes
☒	☐	Estimates of effect sizes (e.g. Cohen's *d*, Pearson's *r*), indicating how they were calculated

Our web collection on statistics for biologists contains articles on many of the points above.

Software and code

Policy information about availability of computer code

Data collection	We used a Qualtrics survey to collect the data of our multi-site study. We did not use any software for the Data collection.
Data analysis	We used Rstudio version 2023.09.0+463 for the data analysis of the current project.

For manuscripts utilizing custom algorithms or software that are central to the research but not yet described in published literature, software must be made available to editors and reviewers. We strongly encourage code deposition in a community repository (e.g. GitHub). See the Nature Portfolio guidelines for submitting code & software for further information.

Data

Policy information about availability of data

All manuscripts must include a data availability statement. This statement should provide the following information, where applicable:

- Accession codes, unique identifiers, or web links for publicly available datasets
- A description of any restrictions on data availability
- For clinical datasets or third party data, please ensure that the statement adheres to our policy

Full data are publicly available at https://osf.io/6w2zm/ and https://github.com/alessandro992/A-large-multi-site-test-of-self-administered-mindfulness/blob/main/finaldata.csv

Research involving human participants, their data, or biological material

Policy information about studies with human participants or human data. See also policy information about sex, gender (identity/presentation), and sexual orientation and race, ethnicity and racism.

Reporting on sex and gender	We determined the gender of participants based on self-reporting methods. Participants could answer to which gender they identified the most, being given six different options (i.e., male, female, transgender male, transgender female, prefer not to say, and an open answer in which they could write their gender). We did not collect disaggregated sex and gender data. We did not conduct sex- and gender-based analyses because the literature we had reviewed did not provide us with evidence to predict gender differences regarding mindfulness and stress reduction.
Reporting on race, ethnicity, or other socially relevant groupings	Participants reported their country of origin and the country where they currently lived. This information was only used to describe the sample; we have not conducted any analysis involving such information.
Population characteristics	See above.
Recruitment	The sites involved in this project recruited participants with a Qualtrics link that was provided to them by the main investigator. The sites' coordinators were told by the main investigator that participants could be recruited using the SONA system of their respective institution or via crowdsourcing platforms such as mTurk or Prolific Academic. While using a combination of unpaid (e.g., SONA) and paid (e.g., Prolific) participation platforms could have mitigated self-selection bias, conducting the experiment solely online still limited our ability to completely eliminate this bias. Just like with practically any randomized controlled trial (RCT) on human subjects, self-selection of participants is inevitable. Participants, patients, etc., can decide whether they want to take part in the study. They can do so before and at any time during the experiment. Self-selection is critical when the goal is to describe a population (e.g., prevalence studies). It is, however, not a threat to the integrity of the results when the goal is to establish causal knowledge because, by definition, causal inference in RCTs is comparative, where we want to examine evidence for relative treatment effectiveness (Msaouel et al., 2023). The goal of an RCT is thus not to arrive at particular statements about the current state of the population, but rather identify and disentangle causal mechanisms. These principles are then likely transportable to the members of the given population, and frequently even beyond (Bradburn et al., 2020).
Ethics oversight	The study first received ethical approval from Swansea University's School of Psychology Research Ethics Sub-committee, while the sites that participated in the data collection either received ethical approval from their local IRBs or stated that they were exempt (e.g., if their IRB accepted the ethics approval awarded by Swansea University and did not request the local collaborator to submit their own application). Each site's IRB protocols with ethics details and acceptance of each protocol can be found on the OSF project page at https://osf.io/6w2zm/. Swansea University and Université Grenoble Alpes carried the administrative organization for the study. Swansea University was also the data controller for this project. The personal data of participants were processed for the purposes outlined in the information sheet (see the document Information Sheet at https://osf.io/xuznc/). Standard ethical procedures involved participants providing their consent to participate in this study by completing the consent form that was administered at the beginning of the online survey used for the experiment.

Note that full information on the approval of the study protocol must also be provided in the manuscript.

Field-specific reporting

Please select the one below that is the best fit for your research. If you are not sure, read the appropriate sections before making your selection.

☐ Life sciences ☒ Behavioural & social sciences ☐ Ecological, evolutionary & environmental sciences

For a reference copy of the document with all sections, see nature.com/documents/nr-reporting-summary-flat.pdf

Behavioural & social sciences study design

All studies must disclose on these points even when the disclosure is negative.

Study description	The current multi-site project followed the route of a parallel randomized controlled trial.
Research sample	The study included participants from Australia, Europe, the UK, Canada, and the US. We had three exclusion criteria:<br>1) Participants had to be current non-meditators or to have not meditated in the 6 months prior to the experiment,<br>2) Participants had to be fluent or native English speakers, and<br>3) Participants had to declare they had not had a history of mental illness.<br>Criterion 1 was used because the experiment focused on the effects of single brief exercises on non-meditators to better understand the potential benefits of mindfulness practices for this population. Criterion 2 was used because the audio files used were recorded in English. Criterion 3 was used because previous research has shown that mindfulness interventions have at times resulted in psychotic episodes, panic attacks, and depersonalization; thus, we needed to screen out participants for whom the mindfulness intervention could have been detrimental.

The final sample was not representative. After excluding participants that did not fit our inclusion criteria, we retained 2,239 valid observations (of these, 611 self-identified as males, 1,576 as females, seven as transgender males, two as transgender females, 27 did not identify with any choice, 16 preferred not to say; Mage = 22.4, SDage = 10.1; range 17-87; 94.2% students).
The rationale behind selecting the study sample (i.e., participants who were non-meditators, fluent in English and had no history of mental illness) was to specifically examine the effects of brief self-administered mindfulness interventions in isolation from potential confounds. A previous experience of engaging with meditation could have changed the baseline for any potential mindfulness effects; non-fluent English language proficiency could have created problems with mindfulness instruction comprehension; and a history of mental illnesses might have exacerbated potential meditation-related adverse effects (Britton, Lindahl, Cooper, Canby & Palitsky, 2021). This study aimed to deepen our understanding of how mindfulness practices can benefit individuals who are new to mindfulness. Although the sample is not representative of the general population, it provides valuable insights into the unique impacts of mindfulness on stress reduction measured experimentally, and practical relevance of mindfulness techniques among a predominantly young, student demographic spread across various geographic regions.

Sampling strategy

Each site collected the data using Qualtrics that redirected participants to the same survey; however, the URL address was tailored for each data-collecting site to allow recording the site participants belonged to. Participants were randomly allocated to the experimental conditions or control using Qualtrics' random block function. Prior to data collection, we conducted a simulation based on a Bayes Factor Design Analysis (BFDA) to assess the expected efficiency and informativeness of our study design. The simulation aimed to determine (1) the expected likelihood of the study to provide compelling evidence either in favor of H0 (BF01 = 1/10) or H1 (BF10 = 10), (2) the likelihood of obtaining convincing but misleading evidence, and (3) the likelihood that the study points in the correct direction even if stopped earlier due to pragmatic constraints on sample size. We modeled a sequential design with a maximum N of 720 and a minimum sample size of 420 participants per condition, with a goal of detecting an effect size of d = 0.20. We tested four interventions using a between-participants adaptive group design and found that the probability of arriving at compelling evidence was .79. A more detailed explanation of this simulation can be found at p. 10 of the manuscript.

Data collection

The experiment was conducted entirely online, and participants were instructed to complete it in a quiet environment for 20 minutes. Participants were asked to access the experiment using a desktop or laptop computer and not a mobile smartphone because one condition of the experimental design involved mindful walking, so we needed to ensure that any participant would be able to complete that task, if they were (randomly) distributed in that condition. The researchers were blind to the experimental conditions the participants were allocated to because the allocation was done using Qualtrics' randomizer function.

Timing

The data collection started on March 23rd, 2022 and finished on June 30th, 2022.

Data exclusions

The dataset originally comprised 6,691 responses, including both the 'test answers' generated by the site collaborators while developing and previewing the survey, and the actual answers submitted by the participants. From the survey's initial participants, we excluded the following: 1,307 who self-identified as meditators or reported having engaged in meditation within six months prior to the experiment, 776 who did not meet the English language proficiency requirement, and 981 who disclosed having a history of mental illnesses. Finally, 1660 participants started the survey without using a smartphone with headphones attached. Among these participants who failed to meet the inclusion criteria, 1,491 simultaneously met multiple exclusion criteria. Respondents who did not meet one or more inclusion criteria (N = 3, 233) were immediately directed towards the end of the survey, and we did not record further data from them. We also removed from analyses those who initiated the survey but did not progress up to the listening of the audio track (N = 976), and the 'test answers' provided by the collaborating researchers while developing the survey (N = 19); thus, the sample size dropped to N = 2,463. We then removed data from 19 participants that dropped out of the experiment and data from 205 participants who, according to our criteria, were considered careless respondents, yielding a final sample of 2,239 valid observations. Of these, 611 participants self-identified as male, 1,576 as female, seven as transgender male, two as transgender female, 27 did not identify with any choice, and 16 preferred not to say (Mage = 22.4, SDage = 10.1; range 17-87; 94.2% students), with an approximately even distribution across the five experimental conditions (Nmindful walking = 416, Nmindful breathing = 469, Nloving - kindness = 427, Nbody scan = 449, Nbook chapter – control = 478). We are not aware of how many participants were invited to the survey, but declined to participate.

Non-participation

We did not collect data from participants who declined to provide consent, as they were led to the end of the survey. However, participants who began the experiment but dropped out before the set of responses related to the main dependent variable were categorized as "careless participants" and were excluded from the main analyses. We do not have information regarding the reasons why participants may have abandoned the experiment.

Randomization

Participants were randomized to one of the experimental conditions (1,2,3,4) or to one of the active control conditions (story a,b,c).
As an example involving 15 participants,this is how they were expected to be randomized by the Qualtrics software:
Condition 1 Body-scan : 3 participants
Condition 2 Loving kindness: 3 participants
Condition 3 Mindful breathing: 3 participants
Condition 4 Mindful walking: 3 participants
Condition 5 control condition story a: 1 participant
Condition 5 control condition story b: 1 participant
Condition 5 control condition story c: 1 participant

Reporting for specific materials, systems and methods

We require information from authors about some types of materials, experimental systems and methods used in many studies. Here, indicate whether each material, system or method listed is relevant to your study. If you are not sure if a list item applies to your research, read the appropriate section before selecting a response.

Materials & experimental systems

n/a	Involved in the study
☒	☐ Antibodies
☒	☐ Eukaryotic cell lines
☒	☐ Palaeontology and archaeology
☒	☐ Animals and other organisms
☐	☒ Clinical data
☒	☐ Dual use research of concern
☒	☐ Plants

Methods

n/a	Involved in the study
☒	☐ ChIP-seq
☒	☐ Flow cytometry
☒	☐ MRI-based neuroimaging

Clinical data

Policy information about clinical studies

All manuscripts should comply with the ICMJE guidelines for publication of clinical research and a completed CONSORT checklist must be included with all submissions.

Clinical trial registration

https://clinicaltrials.gov/study/NCT06308744

Study protocol

The full protocol of the study can be found at https://osf.io/uf4jz (Registration DOI: https://doi.org/10.17605/OSF.IO/UF4JZ) and in our ClinicalTrials.gov page (https://clinicaltrials.gov/study/NCT06308744).

Data collection

Data collection for the study occurred from March 23rd to June 30th, 2022. This study was conducted in a fully decentralized manner, meaning that participants did not visit a laboratory for data collection. Instead, participants engaged with the experiment remotely (e.g., from their home) through a provided Qualtrics link.

Outcomes

For our primary outcome measure, we utilized the 20-item State-Trait Anxiety Inventory, Form Y-1 (STAI19) to evaluate the immediate stress responses of the participants. They were asked to express their current feelings through 20 specific statements, such as "I am tense" and "I feel frightened," employing a 4-point scale ranging from "Not at all" to "Very much so." This scale's utility is underpinned by its established correlation with stress biomarkers, like salivary α-amylase, in prior studies, thereby providing a robust measure of the short-term effects of stress. As a secondary outcome measure, we incorporated the Self-Assessment Manikin scale, a non-verbal, pictorial tool that assesses emotional responses across three dimensions: pleasure, arousal, and dominance. Though not the primary focus of our research, this scale was included for exploratory analysis to enrich our understanding of the participants' emotional states after the listening of the audio track.

Plants

Seed stocks

Report on the source of all seed stocks or other plant material used. If applicable, state the seed stock centre and catalogue number. If plant specimens were collected from the field, describe the collection location, date and sampling procedures.

Novel plant genotypes

Describe the methods by which all novel plant genotypes were produced. This includes those generated by transgenic approaches, gene editing, chemical/radiation-based mutagenesis and hybridization. For transgenic lines, describe the transformation method, the number of independent lines analyzed and the generation upon which experiments were performed. For gene-edited lines, describe the editor used, the endogenous sequence targeted for editing, the targeting guide RNA sequence (if applicable) and how the editor was applied.

Authentication

Describe any authentication procedures for each seed stock used or novel genotype generated. Describe any experiments used to assess the effect of a mutation and, where applicable, how potential secondary effects (e.g. second site T-DNA insertions, mosiacism, off-target gene editing) were examined.
